# Draft genome sequences of seven isolates of *Phytophthora ramorum* EU2 from Northern Ireland

**DOI:** 10.1016/j.gdata.2015.09.009

**Published:** 2015-09-22

**Authors:** Lourdes de la Mata Saez, Alistair R. McCracken, Louise R. Cooke, Paul O'Neill, Murray Grant, David J. Studholme

**Affiliations:** a School of Biological Sciences, Queen's University Belfast, Belfast, Northern Ireland, United Kingdom; b Agri-Food and Biosciences Institute, Belfast, United Kingdom; c Biosciences, University of Exeter, Devon, United Kingdom

**Keywords:** Oomycetes, Phytophthora, Sudden larch death, Phytopathogen

## Abstract

Here we present draft-quality genome sequence assemblies for the oomycete *Phytophthora ramorum* genetic lineage EU2. We sequenced genomes of seven isolates collected in Northern Ireland between 2010 and 2012. Multiple genome sequences from *P. ramorum* EU2 will be valuable for identifying genetic variation within the clonal lineage that can be useful for tracking its spread.

**Table:** 

Specifications	
Organism/cell line/tissue	*Phytophthora ramorum*
Sex	Not applicable
Sequencer or array type	Illumina HiSeq
Data format	Analysed; i.e. raw data was filtered and then assembled.
Experimental factors	Genomic sequence of pure microbial cultures
Experimental features	Genomic sequence of pure microbial cultures
Consent	Not applicable. Data are available without restriction.
Sample source location	Northern Ireland, United Kingdom

## 1 Direct links to deposited data

http://www.ncbi.nlm.nih.gov/bioproject/292010.

http://www.ncbi.nlm.nih.gov/bioproject/292002.

http://www.ncbi.nlm.nih.gov/bioproject/291998.

http://www.ncbi.nlm.nih.gov/bioproject/291995.

http://www.ncbi.nlm.nih.gov/bioproject/291992.

http://www.ncbi.nlm.nih.gov/bioproject/291990.

http://www.ncbi.nlm.nih.gov/bioproject/291989.

## 2 Experimental design, materials and methods

Members of the genus *Phytophthora*, like other oomycetes, superficially resemble fungi but are completely distinct from true fungi both phylogenetically [1] and in their phenotypic characteristics; for example, unlike true fungi, the oomycetes are usually diploid [2]. The genus includes numerous important pathogens of plants, including trees and food crops [3]. The species *Phytophthora ramorum* [4] is an invasive and aggressive pathogen that is believed to have been introduced into North America and Europe [5] from an unknown and possibly Asian centre of origin [6]. Two distinct clonal lineages in North America and a third lineage in Europe appear to have been genetically isolated from each other for tens of millennia [7]. Since 1995 the NA1 lineage and, later, the NA2 lineage have been responsible for the deaths of millions of trees in North America as a result of Sudden Oak Death [8]. The EU1 lineage was originally discovered in Europe on *Rhododendron* and *Viburnum* species in nurseries and gardens [4] and has also crossed the Atlantic to North America [9]. Until recently, *P. ramorum* in Europe was mostly confined to ornamentals in nurseries and gardens but since 2009, *P. ramorum* has been causing landscape-scale epidemics on Japanese larch (*Larix kaempferi*) plantations in the UK [10]. In 2012, the discovery of a fourth clonal lineage of *P. ramorum* was reported; this is currently known to occur only in Northern Ireland and western Scotland [11] and designated as EU2.

We used the Illumina HiSeq to generate 100-bp paired sequence reads from genomic DNA prepared from seven isolates of EU2 collected from multiple sites in Northern Ireland between 2010 and 2012 (Table 1). We filtered and trimmed the reads using TrimGalore (with a threshold score of 30) and performed *de novo* assembly of the trimmed reads using SPAdes 3.5.0 with default parameter values. Finally, we generated scaffolds and performed gap-filling with SSPACE 3.0 [12]. The following options and parameter values were used in SSPACE: -x 1 -m 50 -o 10 -z 200 -p 1.

We estimated the completeness of our assemblies with the CEGMA pipeline [13]. Briefly, CEGMA checks for the presence of each of 248 genes that are ultra-conserved across a range of eukaryotic genomes. It reports how many of these genes are present in the assembled sequence, either as complete genes or partial genes. Based on CEGMA output, it appears that our genome assemblies are slightly more complete than the previously published *P. ramorum* Pr102 genome sequence [14]. However, the total lengths of our assemblies are only about 77% of the length of the published Pr102 genome assembly (Table 2). This likely reflects the limitations of our simple approach involving sequencing of a single short-insert with a short-read sequencing platform in contrast with the Pr102 genome project, which used Sanger chemistry. In both the present study and the Pr102 project, a whole-genome shotgun approach was used.

Genome sequences have been invaluable in the study of *Phytophthora* plant pathogens [15]. Until now, genome sequence data have only been published for the EU1 lineage of *P. ramorum* [14]. Availability of genome sequence data from the EU2 lineage will be a useful resource for investigating the relationships among the four lineages as well as for developing assays for detection and monitoring. Multiple genome sequences from *P. ramorum* EU2 will be valuable for identifying genetic variation within the clonal lineage that can be useful for tracking its spread [16], [17]; no microsatellite sequence variation was known among the originally described isolates of EU2 [11], but the availability of genomic sequence data will facilitate the development of molecular markers based on microsatellites and/or single-nucleotide variants.

## Figures and Tables

**Table 1:** Sequenced isolates.

Isolate	Host	Date of isolation
SOD 158/11	Larch	2011
SOD 58/12	Noble-fir	2012
SOD 69/12	Larch	2012
SOD 22/12	*Rhododendron* species	2012
SOD 136/11	Larch	2011
SOD 169/11	*Rhododendron* species	2011
SOD L51	*Rhododendron* species	2010

**Table 2:** Assembly statistics for the genome sequences reported in this study compared with those for the previously sequenced *P. ramorum* Pr102 [14].

Genome	Accession numbers	Contig assembly size (bp)	Number of contigs	Contig N_50_ (bp)	CEGMA coverage: % complete (% partial)
SOD 158/11	LHTR01000000	41,321,513	5100	23,395	93.95 (97.18)
SOD 58/12	LHTS01000000	41,965,240	4817	27,285	93.55 (97.18)
SOD 69/12	LHTT01000000	41,749,343	4661	27,831	93.95 (97.58)
SOD 22/12	LHTU01000000	41,724,146	4906	26,514	93.55 (97.18)
SOD 136/11	LHTV01000000	41,815,862	4600	27,950	93.55 (96.77)
SOD 169/11	LHTW01000000	41,958,963	4719	27,788	93.95 (97.58)
SOD L51	LHTX01000000	41,834,411	4806	25,735	94.35 (97.58)
Pr102	AAQX01000000	54,424,978	7589	47,511	92.34 (95.16)
